# Interplay between key metabolic hormones, metabolic factors, renal function, and heart rate variability in humans with obesity

**DOI:** 10.1038/s41598-025-21757-1

**Published:** 2025-10-29

**Authors:** Kitchaya Pongwattanapakin, Chit Care, Chantacha Sitticharoon, Kittikorn Tommy Wilasrusmee, Issarawan Keadkraichaiwat, Pailin Maikaew, Rungnapa Sririwichitchai

**Affiliations:** Department of Physiology, Faculty of Medicine Siriraj Hospital, Mahidol University, 2 Wanglang Rd., Siriraj, Bangkoknoi, Bangkok, 10700 Thailand

**Keywords:** Metabolic syndrome, Insulin resistance, HRV, Leptin, Adiponectin, HDL-C, Metabolic syndrome, Obesity, Metabolic syndrome, Obesity

## Abstract

This study aimed to provide the first integrative assessment of clinical, metabolic, renal, hormonal, and heart rate variability (HRV) parameters in individuals with obesity, stratified by metabolic syndrome (MetS) and insulin resistance (IR), clarifying shared and distinct mechanisms beyond prior HRV- or hormone-focused studies. Among 45 participants with obesity (BMI ≥ 25 kg/m²), 67% had IR and 42% had MetS. Both groups exhibited increased %fat, triglyceride, leptin, and resting heart rate, with decreased QUICKI and high-density lipoprotein cholesterol (HDL-C) (*p* < 0.05 all). Frequency-domain HRV (VLF and LF ms²) and overall variability (SDNN) were significantly decreased in the IR group (*p* < 0.05 all). Leptin showed significant positive correlations with obesity, IR, and creatinine clearance (*p* < 0.05 all). Adiponectin exhibited positive correlations with hip circumference, HDL-C, and pNN50 while HDL-C showed negative correlations with the number of MetS criteria, obesity, IR, and leptin, but positive correlations with parasympathetic HRV (SDSD and RMSSD) (*p* < 0.05 all), suggesting a protective role across multiple systems. Creatinine clearance and eGFR revealed positive correlations with parasympathetic HRV (HF nu) and negative correlations with sympathetic HRV (LF nu and LF/HF ratio). In conclusion, this study underscores the complex interplay between these systems, enhancing our understanding of their shared and distinct mechanisms.

## Introduction

Obesity is strongly associated with metabolic syndrome (MetS), insulin resistance (IR), cardiac dysfunction, altered heart rate variability (HRV)^1^, and impaired kidney function^2^, reflecting its broad impact on multiple systems. MetS is characterized by a complex combination of metabolic abnormalities, including central obesity, IR, hypertension, and dyslipidemia^3,4^. MetS affects approximately 20–25% of the adult population worldwide, and these abnormalities pose a significant risk for the development of type 2 diabetes mellitus (DM) and atherosclerotic cardiovascular diseases^5^. The etiology of MetS is multifactorial, with the accumulation of fatty tissue in the abdomen (central obesity) playing a key role, and IR is considered the core feature of the syndrome^5^. IR is defined as the impaired response of target tissues to insulin-mediated glucose disposal, which triggers compensatory hyperinsulinemia, leading to the development of MetS, type 2 DM, and other metabolic disorders, including cardiovascular disease and non-alcoholic fatty liver disease^6,7^.

MetS is not only associated with IR but also with several conditions, such as hypertension, dyslipidemia, chronic inflammation, and autonomic dysfunction^8–10^. Previous studies have reported that autonomic dysregulation is associated with metabolic abnormalities in humans^9,10^. Autonomic cardiac regulation can be assessed by a non-invasive technique called HRV, which is a crucial indicator of cardiovascular health and adaptability to stressors^11^.

HRV refers to the variation in the time intervals between successive heartbeats, comprising time- and frequency-domain metrics^12,13^. Time-domain HRV determines the amount of variability in measurements of the inter-beat interval, including the standard deviation of all NN intervals (SDNN), which is associated with total autonomic activity, the standard deviation of differences between adjacent NN intervals (SDSD), the square root of the mean of the sum of the squares of differences between adjacent NN intervals (RMSSD), and the percentage of intervals differing by more than 50 milliseconds (pNN50)^13^, which reflect parasympathetic activity^11,14,15^. Frequency-domain HRV measures the distribution of absolute or relative power into four frequency bands: total power, which reflects the variance of NN intervals over the temporal segment; very low frequency (VLF); low frequency (LF); and high frequency (HF) power^13^. LF and HF power in ms² represent the absolute power in each band, and when expressed in normalized units (nu), they reflect the relative contribution of each component as a proportion of the total power, excluding the VLF component. The LF/HF ratio is considered an indicator of the balance between sympathetic and parasympathetic tone^11,14^.

In a human study, HRV parameters, including time-domain HRV, specifically SDNN, and frequency-domain HRV, specifically HF, LF, and VLF, in individuals with MetS were significantly lower than those in individuals without MetS, implying the negative impact of MetS on HRV^12^. Additionally, another study in humans reported that components of MetS, including hypertension, type 2 DM, and dyslipidemia, exhibited negative correlations with HRV parameters, namely SDNN, HF, and LF^16^. Furthermore, HRV parameters, specifically SDNN, RMSSD, and HF power, were decreased, but the LF/HF ratio was increased in subjects with IR compared to subjects without IR^17,18^.

Given the complexity of MetS and IR, understanding the impact on autonomic functions, hormonal profiles, and kidney health is important. While the effects of MetS and IR on hormonal profiles and HRV have been studied, the intricate relationships between metabolic factors, hormones, kidney functions, and HRV in the context of MetS and IR remain incompletely understood, especially in individuals with obesity. This study aimed to provide the first integrative assessment of clinical, metabolic, renal, hormonal, and HRV parameters in individuals with obesity, categorized by the presence or absence of MetS or IR, and to examine correlations among these factors, thereby revealing shared and distinct characteristics of MetS and IR beyond previous HRV- or hormone-focused studies. Uncovering these insights may provide a deeper understanding of the complex mechanisms underlying metabolic, autonomic, and cardiovascular disorders.

## Results

### Comparisons of clinical parameters and kidney function tests between participants with or without MetS or IR

Comparisons of clinical parameters and kidney function tests between participants with or without MetS or IR are presented in Table 1. The male-to-female ratio was 13:13 in the Non-MetS group and 5:14 in the MetS group, while it was 6:9 in the Non-IR group and 12:18 in the IR group (Table 1). Both before and after sex adjustment, individuals with MetS showed significantly higher fat percentage (%fat) (*p* = 0.010 and *p* = 0.043, respectively), systolic blood pressure (SBP), diastolic blood pressure (DBP), and mean arterial pressure (MAP) (*p* < 0.001 all) (Table 1). In contrast, the trends toward higher age (*p* = 0.069) and fat mass (*p* = 0.067) in individuals with MetS attenuated to non-significance after adjusting for sex (Table 1), suggesting that sex differences partly explained these associations. Waist-to-hip ratio (WHR) showed no significant differences between groups before sex adjustment but became significantly higher in participants with MetS compared to those without MetS after sex adjustment (*p* = 0.026) (Table 1).

**Table 1:**
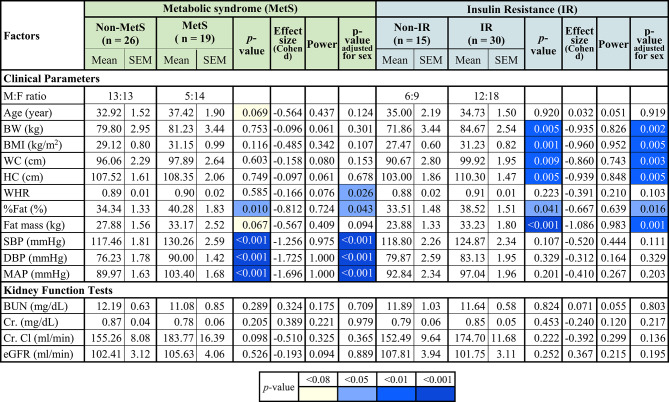
Comparisons of parameters between subjects with non-metabolic syndrome (Non-MetS) and metabolic syndrome (MetS) as well as between non-insulin resistance (Non-IR) and insulin resistance (IR).

*M:F ratio* male-to-female ratio, *BW* body weight, *BMI* body mass index, *WC* waist circumference, *HC* hip circumference, *WHR* waist-to-hip ratio, *%Fat* fat percentage, *SBP* systolic blood pressure, *DBP* diastolic blood pressure, *MAP* mean arterial pressure, *BUN* blood urea nitrogen, *Cr.* Creatinine, *Cr. Cl* creatinine clearance, *eGFR* estimated glomerular filtration rate, *blue shades* significant comparisons, *yellow shade* trends of significant comparisons.

Both before and after sex adjustment, individuals with IR exhibited significantly higher body weight (BW) (*p* = 0.005 and *p* = 0.002, respectively), body mass index (BMI) (*p* = 0.001 and *p* = 0.005, respectively), waist circumference (WC) (*p* = 0.009 and *p* = 0.003, respectively), hip circumference (HC) (*p* = 0.005 both), %fat (*p* = 0.041 and *p* = 0.016, respectively), and fat mass (*p* < 0.001 and *p* = 0.001, respectively) than did individuals without IR (Table 1). Kidney function tests, including blood urea nitrogen (BUN), creatinine (Cr.), creatinine clearance (Cr. Cl), and estimated glomerular filtration rate (eGFR) were not statistically significantly different between participants with or without MetS or IR, either before or after sex adjustment (Table 1).

### Comparisons of metabolic and hormonal profiles between participants with or without MetS or IR

**Fig. 1:**
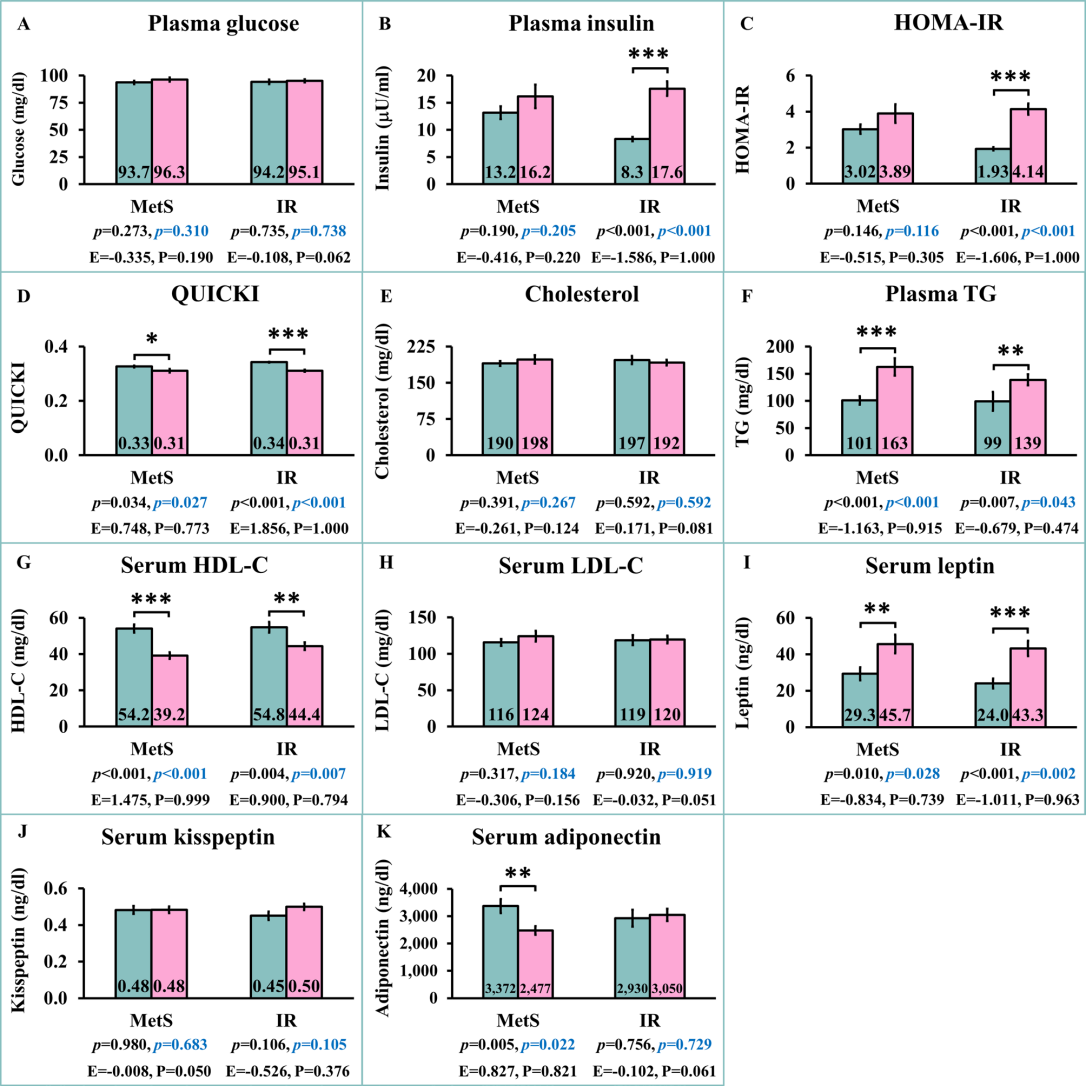
Comparisons of parameters between subjects with non-metabolic syndrome (Non-MetS) and metabolic syndrome (MetS) as well as between non-insulin resistance (Non-IR) and insulin resistance (IR). Green bars = Non-MetS or Non-IR; Pink bars = MetS or IR; black *p* = *p*-values before sex adjustment; blue *p =* *p*-values after sex adjustment; *E* effect size, *P* statistical power. *HOMA-IR* homeostatic model assessment for insulin resistance, *QUICKI *quantitative insulin sensitivity check index, *TG *triglyceride, *HDL-C *high-density lipoprotein cholesterol, *LDL-C *low density lipoprotein cholesterol. *p < 0.05, **p < 0.01, ***p < 0.001 compared between Non-MetS and MetS groups or between Non-IR and IR groups.

Comparisons of metabolic and hormonal profiles between participants with or without MetS or IR are shown in Fig. 1. Both before and after adjusting for sex, plasma triglyceride (TG) (*p* < 0.001 both) (Fig. 1F) and serum leptin (*p* = 0.010 and *p* = 0.028, respectively) (Fig. 1I) were significantly higher, while quantitative insulin sensitivity check index (QUICKI) (*p* = 0.034 and *p* = 0.027, respectively) (Fig. 1D), plasma high-density lipoprotein cholesterol (HDL-C) (*p* < 0.001 both) (Fig. 1G), and serum adiponectin (*p* = 0.005 and *p* = 0.022, respectively) (Fig. 1K) were significantly lower in participants with MetS compared to those without MetS. Both before and after adjusting for sex, plasma insulin (*p* < 0.001 both) (Fig. 1B), homeostatic model assessment for insulin resistance (HOMA-IR) (*p* < 0.001 both) (Fig. 1C), plasma TG (*p* = 0.007 and *p* = 0.043, respectively) (Fig. 1F), and serum leptin (*p* < 0.001 and *p* = 0.002, respectively) (Fig. 1I) were significantly higher, whereas QUICKI (*p* < 0.001 both) (Fig. 1D) and plasma HDL-C (*p* = 0.004 and *p* = 0.007, respectively) (Fig. 1G) were significantly lower in participants with IR compared to those without IR.

### Comparisons of HRV parameters between participants with or without MetS or IR

**Fig. 2:**
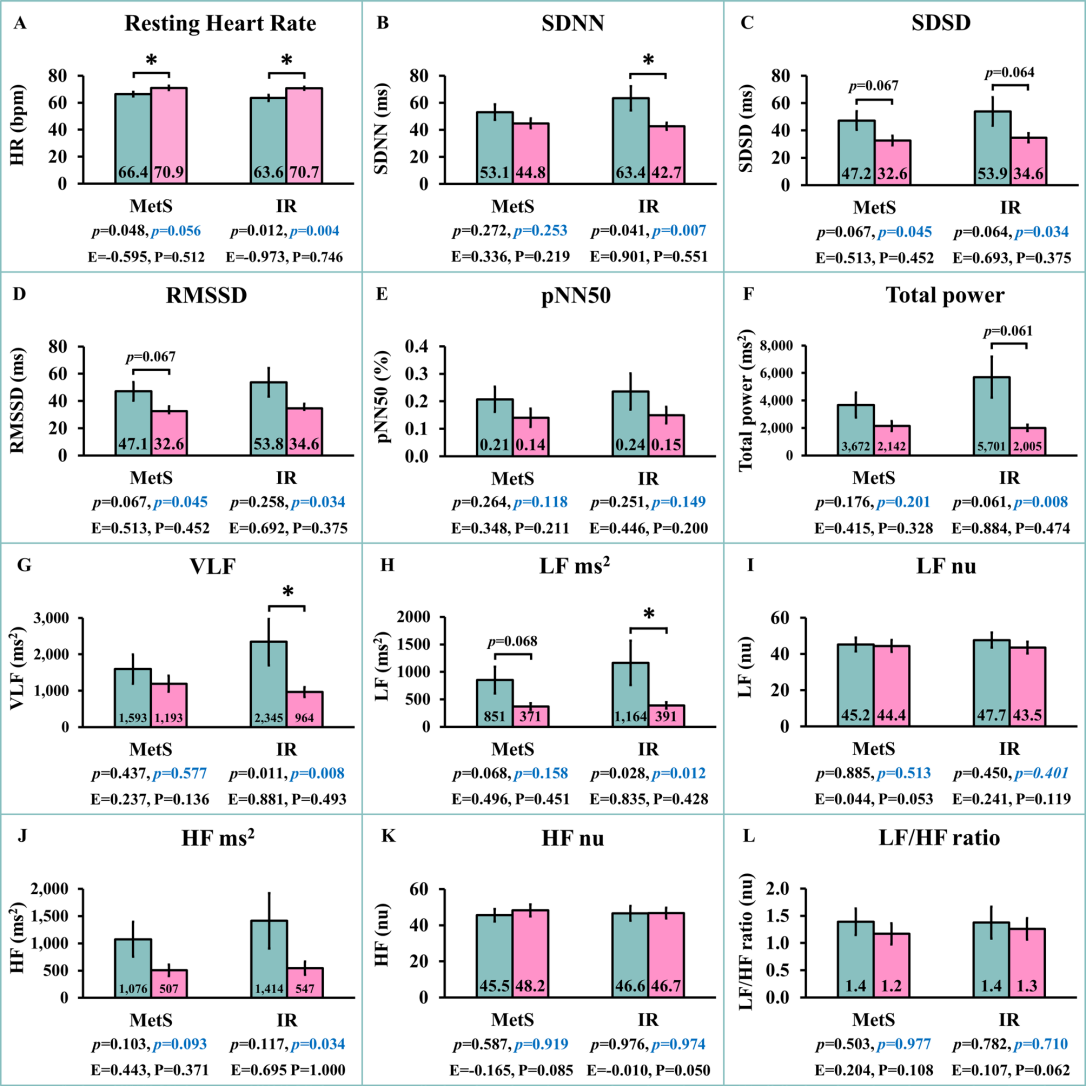
Comparisons of Heart rate variability (HRV) parameters between subjects with non-metabolic syndrome (Non-MetS) and metabolic syndrome (MetS) as well as non-insulin resistance (Non-IR) and insulin resistance (IR). Green bars = Non-MetS or Non-IR; Pink bars = MetS or IR; black *p* = *p*-values before sex adjustment; blue *p* = *p*-values after sex adjustment, *E *effect size, *P *statistical power. *SDNN *standard deviation of all NN intervals, *SDSD* standard deviation of differences between adjacent NN intervals, *RMSSD *the square root of the mean of the sum of the squares of differences between adjacent NN intervals, *pNN50 *percent of pNN50 count, *VLF* power in the very low frequency range, *LF ms*^2^ power in the low frequency range in ms^2^, *LF nu *power in the low frequency range in normalized units, *HF ms*^2^ power in the high frequency range in ms^2^, *HF nu *power in the high frequency range in normalized units, *LF/HF ratio *the ratio of LF ms^2^ to HF ms^2^. *p < 0.05 compared between Non-MetS and MetS groups or between Non-IR and IR groups.

Comparisons of HRV parameters between participants with or without MetS or IR are presented in Fig. 2. In individuals with MetS compared to those without MetS, resting heart rate (HR) was significantly higher before sex adjustment (*p* = 0.048) (Fig. 2A) but became a trend toward higher after sex adjustment (*p* = 0.056). Among MetS participants, trends toward lower SDSD (Fig. 2C) and RMSSD (Fig. 2D) before sex adjustment (*p* = 0.067 both) became significantly lower after sex adjustment (*p* = 0.045 both), while the trend toward lower LF ms² (*p* = 0.068) before sex adjustment became non-significant after sex adjustment (Fig. 2H), indicating that sex differences contributed to these findings.

In IR compared to non-IR individuals, both before and after sex adjustment, resting HR was significantly higher (*p* = 0.012 and *p* = 0.004, respectively) (Fig. 2A), whereas SDNN (*p* = 0.041 and *p* = 0.007, respectively) (Fig. 2B), VLF (*p* = 0.011 and *p* = 0.008, respectively) (Fig. 2G), and LF ms² (*p* = 0.028 and *p* = 0.012, respectively) (Fig. 2H) were significantly lower. In individuals with IR, trends toward lower SDSD (*p* = 0.064) (Fig. 2C) and total power (*p* = 0.061) (Fig. 2F) or non-significantly different HF ms² (*p* = 0.117) (Fig. 2J) before sex adjustment became significantly lower after sex adjustment (*p* = 0.034, *p* = 0.008, and *p* = 0.034, respectively), indicating that parasympathetic reductions were unmasked or strengthened when accounting for sex.

### Correlations between the number of metabolic syndrome criteria, glucose metabolic factors, and lipid profiles with other factors

Correlations between the number of MetS criteria, glucose metabolic factors, and lipid profiles with other factors are presented in Table 2. The number of criteria of MetS was positively correlated with %fat (*p* = 0.001), fat mass (*p* = 0.016), SBP (*p* < 0.001), DBP (*p* < 0.001), plasma insulin (*p* = 0.010), HOMA-IR (*p* = 0.005), plasma TG (*p* < 0.001), Cr. Cl (*p* = 0.042), serum leptin (*p* = 0.006), and resting HR (*p* = 0.011) while exhibited significant negative correlation with QUICKI (*p* = 0.010), HDL-C (*p* < 0.001), and LF ms^2^ (*p* = 0.027) (Table 2). In terms of glucose metabolic factors, plasma insulin and HOMA-IR were positively correlated with BMI (*p* = 0.028 and *p* = 0.025, respectively), HC (*p* = 0.022 and *p* = 0.025, respectively), %fat (*p* = 0.003 and *p* = 0.002, respectively), fat mass (*p* = 0.002 and *p* = 0.001, respectively), Cr. Cl (*p* = 0.003 and *p* = 0.004, respectively), and serum leptin (*p* = 0.003 and *p* = 0.004, respectively) whereas negatively correlated with QUICKI (*p* < 0.001 both) and HDL-C (*p* = 0.003 both). Moreover, resting HR was positively correlated with insulin (*p* = 0.047), showing a trend toward a positive correlation with HOMA-IR (*p* = 0.051). QUICKI showed a positive correlation with HDL-C (*p* = 0.007) but exhibited negative correlations with BW (*p* = 0.002), BMI (*p* = 0.001), WC (*p* = 0.045), HC (*p* = 0.001), %fat (*p* = 0.004), fat mass (*p* < 0.001), plasma insulin (*p* < 0.001),

**Table 2:**
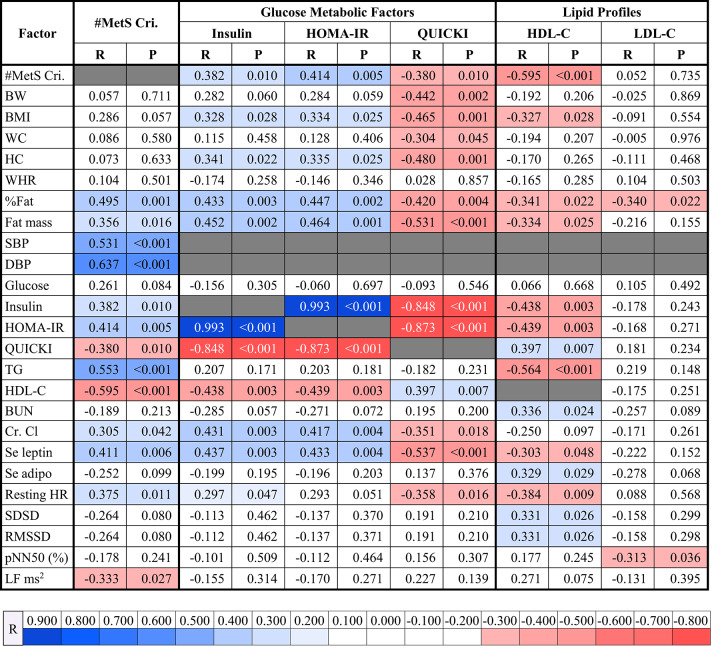
Correlations of number of metabolic syndrome criteria, glucose metabolic factors, and lipid profiles with other factors.

*R* correlation coefficient, *#MetS Cri.* number of metabolic syndrome criteria, *BW* body weight, *BMI* body mass index, *WC* waist circumference, *HC* hip circumference, *WHR* waist-to-hip ratio, *%Fat* fat percentage, *SBP* systolic blood pressure, *DBP* diastolic blood pressure, *HOMA-IR* homeostatic model assessment for insulin resistance, *QUICKI* quantitative insulin sensitivity check index, *TG* triglyceride, *HDL-C* high-density lipoprotein cholesterol, *LDL-C* low density lipoprotein cholesterol, *BUN* blood urea nitrogen, *Cr. Cl* creatinine clearance, *Se* serum, *Adipo* adiponectin, *HR* heart rate, *SDSD* standard deviation of differences between adjacent NN intervals, *RMSSD* the square root of the mean of the sum of the squares of differences between adjacent NN intervals, *pNN50* percent of pNN50 count, *LF ms*^*2*^ power in the low frequency range in ms^2^, *blue shades* significant positive correlations, *red shades* significant negative correlations.

HOMA-IR (*p* < 0.001), Cr. Cl (*p* = 0.018), serum leptin (*p* < 0.001), and resting HR (*p* = 0.016) (Table 2). Regarding lipid profiles, HDL-C was positively correlated with QUICKI (*p* = 0.007), BUN (*p* = 0.024), serum adiponectin (*p* = 0.029), SDSD (*p* = 0.026), and RMSSD (*p* = 0.026) while negatively correlated with BMI (*p* = 0.028), %fat (*p* = 0.022), fat mass (*p* = 0.025), plasma insulin (*p* = 0.003), HOMA-IR (*p* = 0.003), plasma TG (*p* < 0.001), serum leptin (*p* = 0.048), and resting HR (*p* = 0.009), and low-density lipoprotein cholesterol (LDL-C) was negatively correlated with %fat (*p* = 0.022) and pNN50 (*p* = 0.036) (Table 2).

### Correlations between kidney functions and hormones with other factors

Correlations between kidney functions and hormones with other factors are shown in Table 3. BUN was positively correlated with WHR (*p* = 0.006), HDL-C (*p* = 0.024), Cr. (*p* < 0.001), total power (*p* = 0.023), VLF (*p* = 0.009), and LF ms² (*p* = 0.008), while it was negatively correlated with plasma TG (*p* = 0.004), Cr. Cl (*p* = 0.007), eGFR (*p* = 0.002), and resting HR (*p* = 0.017). Cr. exhibited positive correlations with BW (*p* = 0.028), WC (*p* = 0.005), WHR (*p* < 0.001), BUN (*p* < 0.001), LF nu (*p* < 0.001), and LF/HF ratio (*p* = 0.002), but showed negative correlations with %fat (*p* = 0.037), Cr. Cl (*p* < 0.001), eGFR (*p* < 0.001), and HF nu (*p* = 0.001). Cr. Cl was positively correlated with number of MetS criteria (*p* = 0.042), BW (*p* = 0.020), BMI (*p* < 0.001), HC (*p* < 0.001), %fat (*p* < 0.001), fat mass (*p* < 0.001), insulin (*p* = 0.003), HOMA-IR (*p* = 0.004), eGFR (*p* < 0.001), serum leptin (*p* < 0.001), and HF nu (*p* = 0.045), while it was negatively correlated with WHR (*p* = 0.045), QUICKI (*p* = 0.018), BUN (*p* = 0.007), Cr. (*p* < 0.001), LF nu (*p* = 0.010), and LF/HF ratio (*p* = 0.036). eGFR had positive correlations with Cr. Cl (*p* < 0.001) and HF nu (*p* = 0.034), but demonstrated negative correlations with WHR (*p* = 0.008), BUN (*p* = 0.002), Cr. (*p* < 0.001), LF nu (*p* = 0.011), and LF/HF ratio (*p* = 0.028) (Table 3). For hormones, serum leptin was positively correlated with number of MetS criteria (*p* = 0.006), BW, BMI, WC, HC, %fat, fat mass (*p* < 0.001 all), plasma insulin (*p* = 0.003), HOMA-IR (*p* = 0.004), and Cr. Cl (*p* < 0.001), whereas it was negatively correlated with QUICKI (*p* < 0.001) and HDL-C (*p* = 0.048) (Table 3). Serum adiponectin was positively correlated with HC (*p* = 0.014), HDL-C (*p* = 0.029), and pNN50 (*p* = 0.001) (Table 3).

**Table 3:**
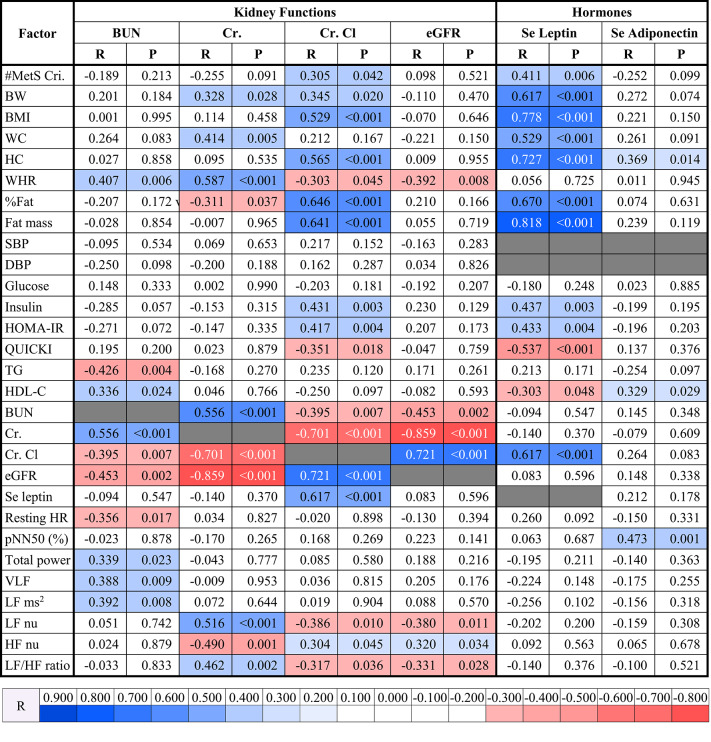
Correlations between kidney functions and hormones with other factors.

*R* correlation coefficient, *#MetS Cri.* number of metabolic syndrome criteria, *BW* body weight, *BMI* body mass index, *WC* waist circumference, *HC* hip circumference, *WHR* waist-to-hip ratio, *%Fat* fat percentage, *SBP* systolic blood pressure, *DBP* diastolic blood pressure, *HOMA-IR* homeostatic model assessment for insulin resistance, *QUICKI* quantitative insulin sensitivity check index, *TG* triglyceride, *HDL-C* high-density lipoprotein cholesterol, *BUN* blood urea nitrogen, *Cr.* Creatinine, *Cr. Cl* creatinine clearance, *eGFR* estimated glomerular filtration rate, *Se* serum, *HR* heart rate, *pNN50* percent of pNN50 count, *VLF* power in the very low frequency range, *LF ms*^*2*^ power in the low frequency range in ms^2^, *LF nu* power in the low frequency range in normalized units, *HF nu* power in the high frequency range in normalized units, *LF/HF ratio* the ratio of LF ms^2^ to power in the high frequency range in ms^2^ (HF ms^2^), *blue shades* significant positive correlations, *red shades* significant negative correlations.

### Multiple regression analysis of the number of metabolic syndrome criteria, serum leptin and adiponectin

The results of the multiple regression analysis of the number of MetS criteria, serum leptin and adiponectin are shown in Table 4. For all regression models, assumptions were satisfied: residuals were approximately normally distributed (histogram and P-P plots; SD = 0.965 for MetS criteria, SD = 0.976 for leptin, SD = 0.976 for adiponectin), and scatterplots indicated linearity and homoscedasticity; collinearity diagnostics confirmed no concern, with tolerance = 0.587–0.931 and VIF = 1.074–1.703 for MetS criteria, Tolerance = 0.935–1.000 and VIF = 1.000–1.069 for leptin, and Tolerance = 0.971 and VIF = 1.030 for adiponectin. In the analysis using the number of MetS criteria as the dependent variable, 4 significant models were observed. The independent variables included DBP (Model 1), or DBP and HDL-C (Model 2), or DBP, HDL-C, and sex (Model 3), or DBP, HDL-C, sex, and TG (Model 4) (*p* < 0.001 all) (Table 4). When sex was removed, %fat emerged as a significant predictor with DBP, HDL-C, and %fat (Model 5), or DBP, %fat, and TG (Model 6), and DBP, HDL-C, %fat, and TG (Model 7) as independent variables (*p* < 0.001 all) (Table 4). This pattern suggests potential collinearity or shared variance between sex and %fat, with sex contributing more unique explanatory power than %fat in predicting the number of MetS criteria. This may be explained by the biological correlation between these variables, sex differences in body composition and metabolic profiles may have allowed sex to function as a proxy for %fat in the model. When serum leptin was set as the dependent variable, 2 models of significant interactions were observed, using either fat mass (Model 1) or fat mass and DBP (Model 2) as independent variables (*p* < 0.001 all) (Table 4). When serum adiponectin was considered as the dependent variable, 2 models with significant interactions were identified, using either HC (Model 1, *p* = 0.014) or HC and HDL-C (Model 2, *p* < 0.001) as independent variables (Table 4). For serum leptin and adiponectin as dependent variables, sex was initially included as a candidate independent variable in the stepwise multiple linear regression models but was excluded during the model selection process, suggesting that sex did not significantly contribute to the variance explained in the final models.

**Table 4:**
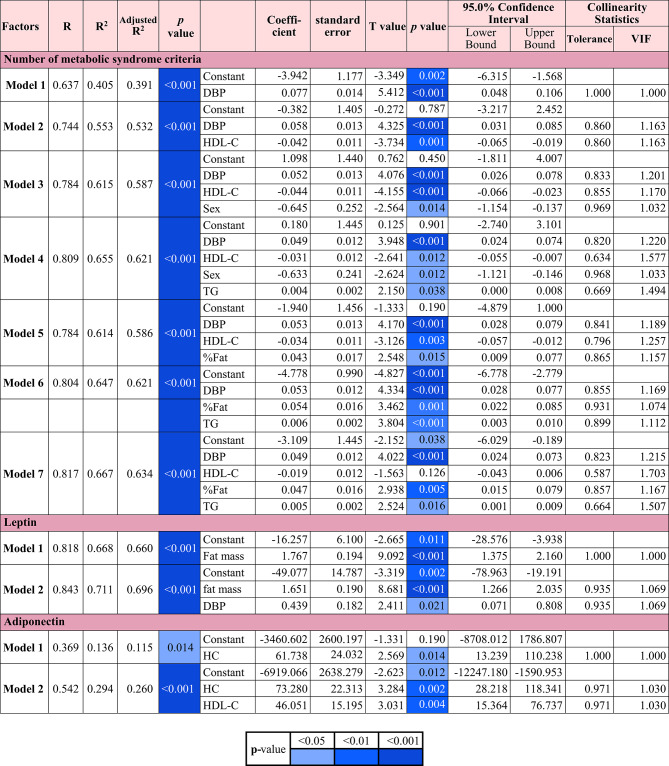
Multiple regression analysis of no. of metabolic syndrome criteria, serum leptin and adiponectin.

*R* correlation coefficient, *DBP* diastolic blood pressure, *HDL-C* high density lipoprotein cholesterol, *Sex* coded as 1 = female, 2 = male, *TG* triglyceride, *%Fat* fat percentage, *HC* hip circumference, *VIF* Variance Inflation Factor, *blue shades* significant positive correlation.

**Fig. 3:**
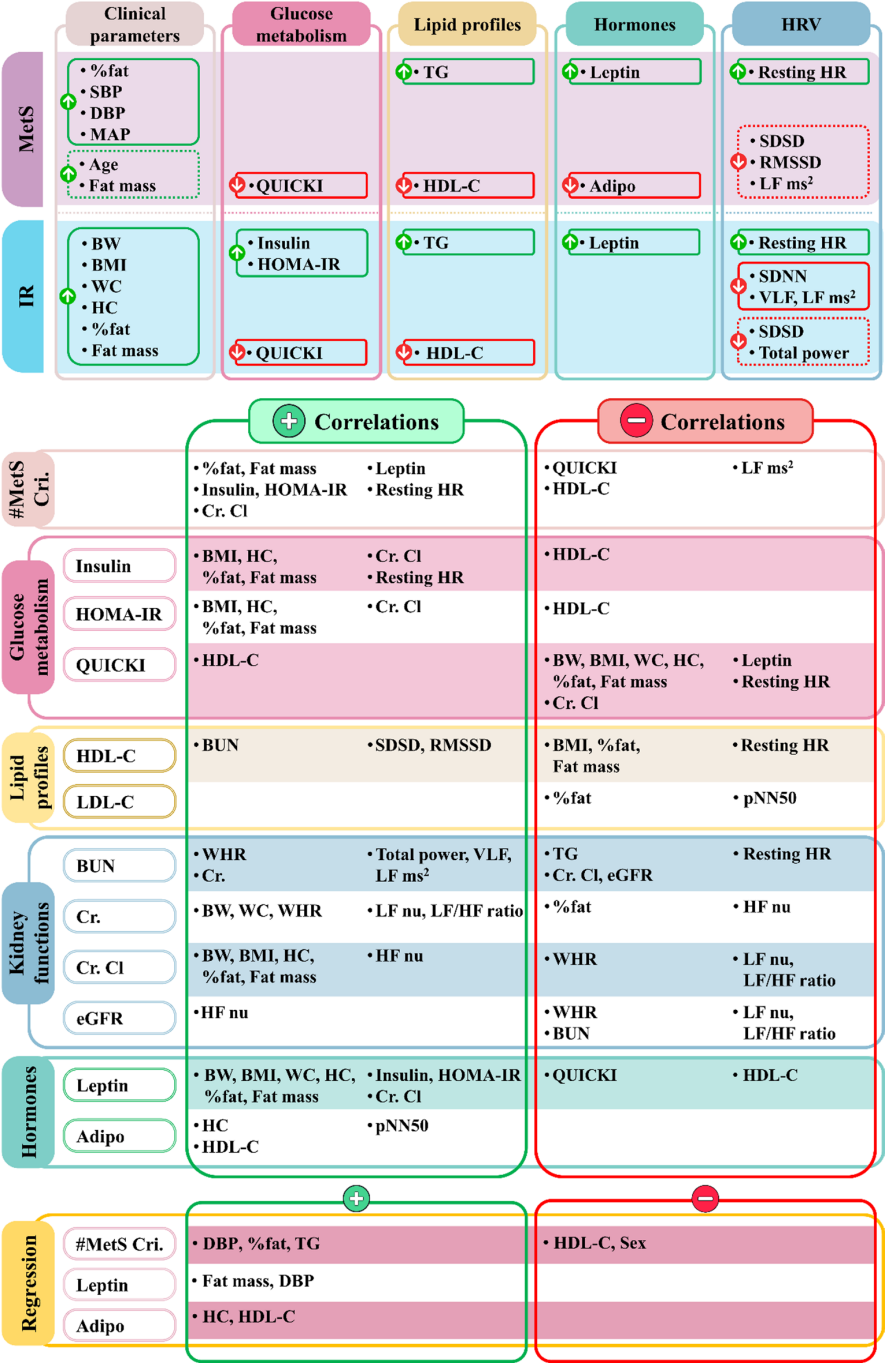
Summary of results. *MetS *metabolic syndrome, *IR *insulin resistance, *#MetS Cri. *number of metabolic syndrome criteria, *%fat *fat percentage, *SBP *systolic blood pressure, *DBP *diastolic blood pressure, *MAP *mean arterial pressure, *QUICKI *quantitative insulin sensitivity check index, *TG *triglyceride, *HDL-C *high-density lipoprotein cholesterol, *LDL-C *low-density lipoprotein cholesterol, *Adipo *adiponectin, *HR *heart rate, *SDSD *standard deviation of differences between adjacent NN intervals, *RMSSD *the square root of the mean of the sum of the squares of differences between adjacent NN intervals, *LF ms*^2^ power in the low frequency range in ms^2^, *BW* body weight, *BMI *body mass index, *WC *waist circumference, *HC* hip circumference, *HOMA-IR *homeostatic model assessment for insulin resistance, *SDNN* standard deviation of all NN intervals, *VLF *power in the very low frequency range, *BUN* blood urea nitrogen, *Cr. *creatinine, *Cr. Cl *creatinine clearance, *eGFR *estimated glomerular filtration rate, *pNN50 *percent of pNN50 count, *WHR *waist-to-hip ratio, *LF nu *power in the low frequency range in normalized units, *HF nu *power in the high frequency range in normalized units, *LF/HF ratio *the ratio of LF ms^2^ to power in the high frequency range in ms^2^ (HF ms^2^); Sex = coded as 1 = female, 2 = male.

The summary of the results is shown in Fig. 3.

## Discussions

**Fig. 4:**
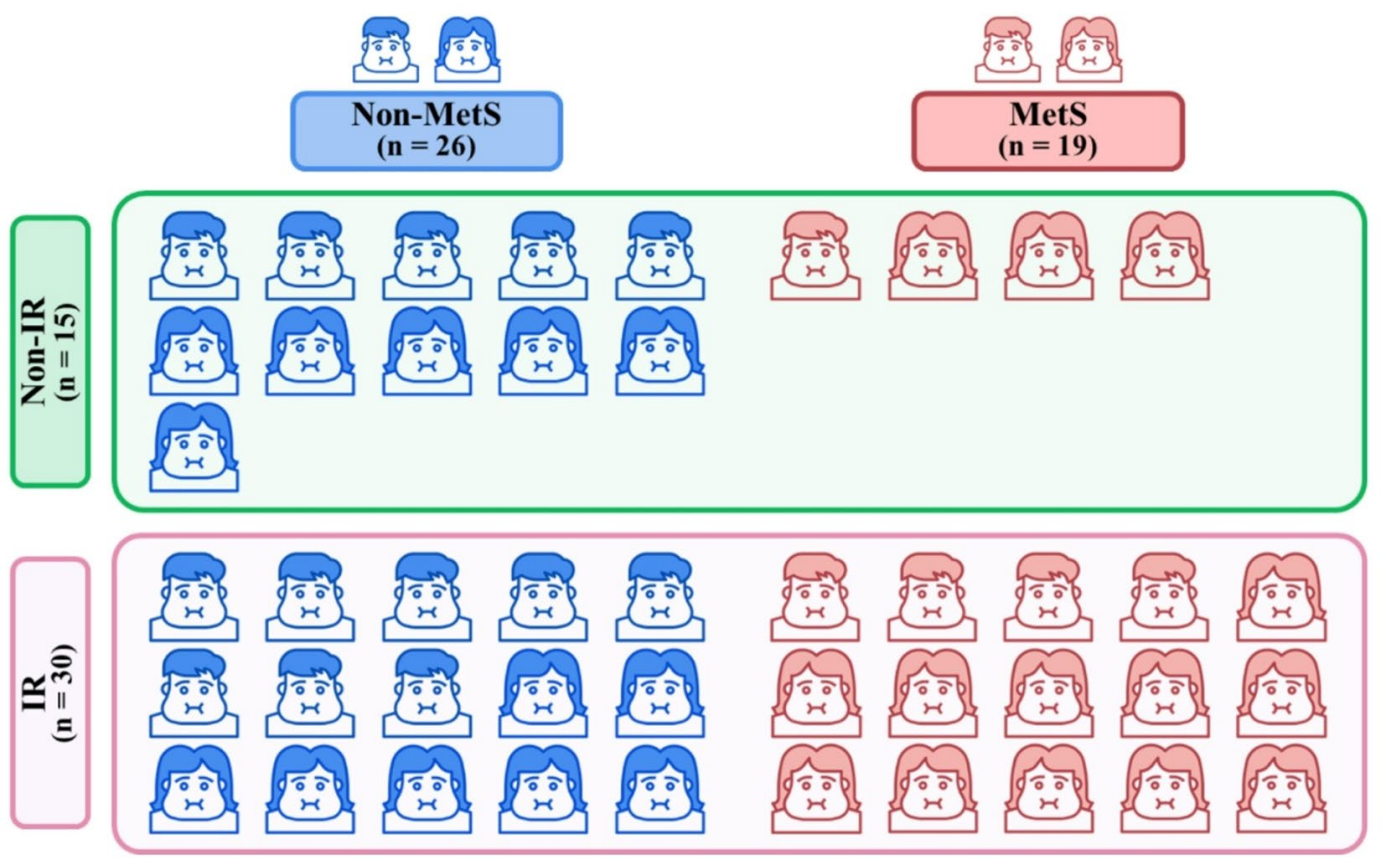
Allocation of subjects. *Non-MetS *participants without metabolic syndrome, *MetS *participants with metabolic syndrome, *Non-IR *participants without insulin resistance, *IR *participants with insulin resistance.

This is the first integrative study of clinical, metabolic, renal, hormonal, and HRV parameters in individuals with obesity, highlighting both shared and distinct mechanisms that may underlie MetS and IR. Among the 45 individuals with obesity, approximately two-thirds had IR, and fewer than half (~ 40%) met the criteria for MetS (Fig. 4). Nearly all individuals (~ 80%) with MetS also had IR, whereas only half of those with IR had MetS. This demonstrates that IR is more prevalent and can occur independently of MetS in obesity (Fig. 4).

Notably, both the MetS and IR groups shared common factors of increased %fat, fat mass, TG, and leptin, along with decreased levels of HDL-C compared to the Non-MetS or Non-IR groups, respectively, indicating increased adiposity and dyslipidemia in both conditions. However, higher plasma insulin and HOMA-IR were observed only in the IR group, suggesting that insulin resistance is prominent in the IR group but not always present in MetS. Higher SBP and DBP and lower serum adiponectin levels were observed only in the MetS group, suggesting that BP and adiponectin may be more specifically associated with MetS rather than IR alone. These findings highlight that, even though both conditions share some common metabolic disturbances, the distinct metabolic profiles of the IR and MetS groups support the idea that these conditions, though overlapping, have unique metabolic features that may influence their clinical manifestations.

Importantly, after adjusting for sex, the MetS-Non-MetS differences showed a mixed pattern: WHR shifted from non-significant to significantly higher in the MetS group, indicating that sex adjustment increased the apparent central adiposity contrast; %fat and SBP, DBP, and MAP remained significant, confirming robust effects; whereas the prior trends for age and fat mass decreased to non-significance, suggesting partial confounding by sex.

The number of MetS criteria was positively correlated with %fat, fat mass, SBP, DBP, plasma insulin, HOMA-IR, TG, Cr. Cl, serum leptin, and resting HR, while it was negatively correlated with QUICKI, HDL-C, and LF ms². Furthermore, resting HR showed a positive correlation with insulin, but negative correlations with QUICKI, HDL-C, and BUN, reflecting that adiposity, blood pressure, insulin resistance, dyslipidemia, kidney function, and HRV were associated with MetS. Among them, DBP, with the strongest correlation to MetS criteria (*R* = 0.637), is influenced by autonomic imbalance, particularly increased sympathetic activity in obesity^19^, that raises both DBP and HR, along with increased vascular resistance^20^ and insulin resistance^21^, together contributing to its central role in MetS. Additionally, the inverse correlation of HDL-C (*R*= - 0.595) with MetS highlights its role in lipid metabolism and endothelial function^22^, further reinforcing the interconnected nature of metabolic and cardiovascular dysfunction in MetS.

Serum levels of leptin, primarily produced in adipose tissues^23^, exhibited positive correlations with the number of MetS criteria, BW, BMI, WC, HC, %fat, fat mass, insulin, and HOMA-IR, highlighting its role in obesity-related metabolic dysregulation and IR. Among these factors, fat mass exhibited the highest correlation coefficient (*R* = 0.818), and was the major contributing factor to leptin levels in regression analysis, which could be predicted by 66.8% (R²=0.668). This aligns with previous dual-energy X-ray absorptiometry-based study showing fat mass as the strongest predictor of leptin^24^, supporting the utility of bioelectrical impedance for estimating leptin in obesity.

The correlation between serum leptin and insulin as well as HOMA-IR could be explained through prolonged β-cell overstimulation by insulinogenic nutrients in people with obesity, causing β-cells’ hyperreactivity to glucose^25^, which could lead to IR. Furthermore, leptin binds to leptin receptors (LEPR) on pancreatic β-cells to inhibit insulin secretion. In individuals with obesity, prolonged high levels of leptin cause leptin resistance, leading to impaired LEPR signaling and disinhibition of insulin secretion in β-cells, and also activate inflammatory pathways (e.g., Cdc42, MAPKs)^26^. Moreover, leptin resistance leads to the reduction in lipid oxidation in insulin-sensitive organs, which may promote lipid accumulation and IR^27^. Taken together, leptin resistance contributes to disrupted insulin regulation and induction of IR.

Interestingly, the number of MetS criteria exhibited a positive association with Cr. Cl. Furthermore, Cr. Cl showed positive correlations with BW, BMI, HC, %fat, fat mass, plasma insulin, HOMA-IR, and serum leptin, while negative correlations with WHR and QUICKI, emphasizing the link between MetS and kidney function through adiposity and IR. Both obesity and IR can induce glomerular hyperfiltration due to increased sympathetic activity^28^ and elevated leptin levels^25^, resulting in an increase in Cr. Cl. Our finding is consistent with previous study in young white males reporting a gradual increase in absolute Cr. Cl across the accumulation of MetS risk factors^29^. Nevertheless, because body weight is incorporated into the calculation of Cr. Cl, its associations with adiposity and metabolic markers may be partly driven by this confounding factor, and results should be interpreted with caution.

This positive relationship between Cr. Cl and serum leptin likely results from the action of leptin in sympathetic activation^30,31^, natriuresis^32^, and induction of nitric oxide (NO) production^33^. Leptin increases sympathetic nerve activity via postsynaptic synergism of glutamatergic inputs onto paraventricular nucleus pre-sympathetic neurons^34^, leading to increased efferent vasoconstriction^35^; induces NO production, causing vasodilation in renal afferent arterioles locally^33^; and has a natriuretic effect that increases sodium excretion and urine volume^36^. Together, these effects increase the filtering surface area and enhance the permeability of the glomerular hydraulic barrier, resulting in elevated Cr. Cl. Our results are consistent with a previous study reporting a positive correlation between Cr. Cl and leptin in patients with continuous ambulatory peritoneal dialysis^37^. Notably, our results showed leptin was only associated with Cr. Cl, but not eGFR, which might be explained by the fact that Cr. Cl, not eGFR, includes BW and is based on 24-hour urine collection and urine creatinine excretion.

Cr. Cl and eGFR showed negative correlations with WHR, whereas BUN and Cr. exhibited positive correlations. These patterns suggest that central obesity may contribute to renal dysfunction over time^2^, possibly through inflammation, IR, and endothelial dysfunction^38^ while also being linked to increased protein catabolism^39^. Among the renal factors, only BUN showed a positive correlation with HDL-C and a negative correlation with TG, which is consistent with a previous study in patients with DM^40^, implying BUN is more related to lipid profiles than other renal factors in obesity.

Serum adiponectin exhibited positive correlations with HC and HDL-C, and regression confirmed both as independent predictors. The positive correlation between adiponectin and HC suggests that adiponectin is more specifically affected by peripheral fat distribution^41,42^. Furthermore, the positive association between adiponectin levels and HDL-C is consistent with previous studies in individuals with increased adiposity^43^ and in those with type 2 DM^44^. This relationship may arise from adiponectin’s ability to increase HDL-C by reducing hepatic lipase expression, enhancing lipoprotein lipase expression^45^, and stimulating the production of ATP-binding cassette transporter A1 (ABCA1), which facilitates HDL-C assembly through reverse cholesterol transport^46^.

HDL-C showed positive correlations with QUICKI, BUN, and serum adiponectin, while negative correlations with the number of MetS criteria, obesity parameters (BMI, %fat, and fat mass), IR parameters (plasma insulin and HOMA-IR), plasma TG, and serum leptin. These results indicate that HDL-C was positively associated with beneficial health parameters but negatively correlated with adverse health parameters. Our results are consistent with prior studies demonstrating that HDL-C promotes adiponectin expression in the abdominal fat^47^ and had a strong positive correlation with plasma adiponectin levels^48^. Besides, a previous study reported that low HDL-C levels are associated with elevated pro-inflammatory cytokines^49^ and HDL-C can suppress inflammatory responses^50,51^.

Furthermore, HDL-C was positively correlated with parasympathetic HRV indices, including SDSD and RMSSD, while it was negatively correlated with resting HR. These findings are consistent with prior research demonstrating that HDL-C was positively associated with parasympathetic indices (HF nu, SDNN, RMSSD, and pNN50)^52,53^, while it was negatively correlated with indices of sympathetic predominance (LF/HF and LF nu)^52–54^. Such correlations suggest that higher HDL-C levels might promote parasympathetic modulation and improve overall HRV, consistent with its known cardioprotective role. Collectively, HDL-C has protective roles across cardiovascular, metabolic, and autonomic systems.

Conversely, LDL-C was negatively correlated with pNN50, a parasympathetic index, highlighting its adverse impact on autonomic regulation. This finding is consistent with previous studies showing that LDL-C was negatively associated with parasympathetic parameters, including RMSSD, pNN50, and HF^54,55^, but was positively correlated with LF/HF ratio^56^, further emphasizing the detrimental impact on parasympathetic activity.

Autonomic dysregulation was observed in both MetS and IR, as evidenced by reductions in both sympathetic HRV components (trends of LF ms² for MetS, and VLF and LF ms² for IR) and parasympathetic HRV components (trends of SDSD and RMSSD for MetS, and SDNN and trends of SDSD and total power for IR). More HRV measures were reduced in the IR group than in the MetS group, indicating that autonomic dysregulation in IR affected a wider range of HRV parameters. It might be explained by the fact that hyperglycemia due to IR causes neuropathy^57^, as well as hyperinsulinemia induces β-adrenergic stimulation^58^, which together lead to an imbalance in autonomic regulation. Nonetheless, autonomic disturbances may be less extensive or influenced by other metabolic factors, such as dyslipidemia and hypertension, which may explain why the MetS group showed fewer reduced HRV parameters. After adjusting for sex, the differences in SDNN, VLF, and LF ms^2^ in the IR group remained significant; however, SDSD and RMSSD became significantly lower in both the MetS and IR groups, whereas total power and HF ms^2^ became significantly lower in the IR group, suggesting that sex-related differences may have obscured underlying parasympathetic dysregulation. This reinforces the importance of controlling for sex in HRV studies, particularly when investigating populations with metabolic abnormalities.

The increased resting HR in both the MetS and IR groups suggests a more pronounced reduction in parasympathetic activity than sympathetic activity, leading to sympathetic dominance. The number of MetS criteria was negatively correlated with LF ms², which is predominantly reflective of sympathetic activity in short-term recordings, suggesting that the more MetS criteria are present, the greater the decrease in HRV, specifically in LF ms². Our study aligns with a previous study reporting that a one-unit increment in the number of MetS risk factors reduces LF by 15%^59^. This highlights a reciprocal relationship between metabolic burden and autonomic tone abnormalities, with reduced HRV.

Regarding kidney function, BUN and Cr. exhibited positive correlations with sympathetic indices (VLF and LF ms² for BUN, and LF nu and the LF/HF ratio for Cr.). BUN also showed a positive correlation with the parasympathetic marker (total power), whereas Cr. had a negative correlation with the parasympathetic index (HF nu), indicating that BUN may reflect compensatory parasympathetic responses, while Cr. is linked to impaired parasympathetic regulation. Our results are in agreement with previous studies showing that Cr. was negatively correlated with SDNN, HF, and total power^53,60^, suggesting that elevated Cr. levels may shift autonomic balance.

Furthermore, eGFR was positively correlated with HF nu and negatively correlated with LF nu and the LF/HF ratio, suggesting that increased eGFR is associated with increased parasympathetic and decreased sympathetic outflow. This study is consistent with a previous study showing that eGFR was positively associated with SDNN, an index of overall HRV with strong parasympathetic influence in short-term recordings^53^. These findings imply that impaired renal function potentially contributes to autonomic dysregulation.

Serum adiponectin showed a positive correlation with pNN50, highlighting its role in promoting parasympathetic activity. Our findings align with previous studies, which reported that adiponectin was positively associated with SDNN and RMSSD in individuals with type 2 DM^61^, and HF in proximal atrial fibrillation^62^. These findings suggest that higher adiponectin levels may contribute to improved autonomic regulation and potentially offer cardiovascular protection^63^.

Beyond metabolic and hormonal pathways, obesity-induced gut dysbiosis may modulate the gut–liver–autonomic axis and contribute to metabolic disturbances^64^. Altered microbial composition and increased gut permeability allow lipopolysaccharides (LPS) from bacterial cell walls to enter circulation^65^, where they activate microglia and trigger neuroinflammation in the hypothalamus-pituitary-adrenal axis^66,67^, reinforcing autonomic imbalance. In parallel, hypoxic adipocytes in obesity secrete pro-inflammatory cytokines (TNF-α, IL-1β, IL-6), driving chronic low-grade inflammation^27^ that underlies complications such as IR and autonomic dysfunction. These inflammatory cytokines inversely correlate with HRV indices (e.g., RMSSD and HF), particularly with vagal modulation^68,69^ as obesity impairs the vagal anti-inflammatory reflex^70^ and weakens parasympathetic tone. Collectively, obesity-related gut dysbiosis and inflammation converge to disrupt autonomic regulation in MetS and IR, linking immune–metabolic dysregulation to elevated cardiovascular risk.

This study has several limitations. First, the unequal male-to-female ratio between the Non-MetS and MetS groups, as well as between the Non-IR and IR groups, with a predominance of females, may reflect the higher prevalence of obesity among females (21%) compared with males (14%) in Asia, including Thailand in 2023 (about 50% higher)^71^. This imbalance may have influenced obesity, blood pressure, lipid, and hormonal parameters, potentially due to the effects of sex hormones. To address this, we performed sex adjustment in both group comparisons and regression analyses. Second, HRV was measured over a 5-minute period (short term), which is widely accepted and commonly used to assess autonomic function in experimental and clinical settings^13^. Short-term recordings provide reliable estimates of parasympathetic and sympathetic activity, particularly for time-domain and high-frequency indices^11,13^. However, they may not capture circadian fluctuations or the full extent of autonomic variability. By contrast, 24-hour monitoring provides more valid measurements of VLF, total power, and LF/HF indices^72^, and yields stronger prognostic value for SDNN^13^. Thus, while short-term HRV was appropriate for the controlled design of our study, 24-hour recordings could offer more comprehensive and precise insights into long-term autonomic regulation.

## Conclusions

Our study demonstrated that among individuals with obesity, IR was more prevalent (67%) than MetS (42%), with considerable but incomplete overlap between the two conditions. Both MetS and IR shared metabolic disturbances, including increased adiposity, TG, leptin, and resting HR, along with decreased HDL-C, QUICKI, and parasympathetic and sympathetic HRV indices. Despite these overlaps, the groups exhibited distinct pathophysiological features, with higher blood pressure and lower adiponectin specific to MetS, whereas greater obesity and insulin resistance parameters characterized IR. Leptin was positively correlated with obesity, IR, and Cr. Cl, indicating systemic involvement, whereas adiponectin and HDL-C were linked to cardioprotective and parasympathetic activity. Kidney function also showed associations with HRV, suggesting a role in autonomic dysregulation. Together, these findings highlight the complex interplay between metabolic, cardiovascular, autonomic, and renal systems in obesity, underscoring both shared and distinct mechanisms in MetS and IR. Future research should investigate whether obesity prevention and management strategies can improve these interrelated pathways, thereby reducing cardiometabolic risk.

## Materials and methods

### Ethics statement

The human study was approved by the Siriraj Institutional Review Board of the Faculty of Medicine Siriraj Hospital, Mahidol University (COA. Si 563/2015) according to international guidelines for human research protection, such as the CIOMS Guidelines, the Declaration of Helsinki, the International Conference on Harmonization in Good Clinical Practice (ICH-GCP), and the Belmont Report. All participants signed informed consent forms prior to the study.

### The study protocols and participants

This cross-sectional study was conducted at the Department of Physiology, Faculty of Medicine Siriraj Hospital, Mahidol University, Thailand, from October 2015 to May 2019. Thai individuals with obesity aged over 18 years with a BMI ≥ 25 kg/m^2^ (criteria for an Asian population)^73^, which is different from the BMI classification for populations of European descent^74^, were recruited into the study. To classify as MetS, an individual must present with 3 or more metabolic abnormalities according to the National Cholesterol Education Program ATP3 2005 guidelines^75^ as follows: a WC > 90 cm in men and > 80 cm in women for Asian populations^76^; plasma TG ≥ 150 mg/dL; plasma HDL-C < 40 mg/dL for men and < 50 mg/dL for women; fasting plasma glucose ≥ 100 mg/dL; and SBP ≥ 130 mmHg or DBP ≥ 85 mmHg. IR was defined as a HOMA-IR > 2.3 in this study, based on non-diabetic populations from the Middle East and Southeast Asia, where values typically range from 1.8 to 2.3^77,78^, and similar to our previous study^79^. Male participants were eligible to take part in the study on any available day, while female participants were required to participate between days 1–3 of their menstrual cycle, during the early proliferative phase, to minimize the confounding effects of sex hormones. Exclusion criteria involved subjects with metabolic diseases, and diseases causing secondary obesity; use of weight-loss medications; regular exercise (defined as at least three 30-minute sessions per week)^80^; and conditions such as menopause, pregnancy, and lactation. Participants with obesity were recruited under strict exclusion criteria to eliminate the confounding effects of metabolic comorbidities. Since many individuals with obesity also present with conditions such as hypertension and diabetes mellitus, the exclusion of these comorbidities limited the number of eligible participants, thereby contributing to the modest sample size. The study included a total of 45 participants, categorized based on either the presence or absence of MetS or IR. The Non-MetS group consisted of 26 individuals, of whom 11 were Non-IR (5 males and 6 females) and 15 were classified as IR (8 males and 7 females). The MetS group consisted of 19 individuals, of whom 4 were Non-IR (1 male and 3 females) and 15 were IR (4 males and 11 females). Allocation of participants in this study is shown in Fig. 4.

### Demographic and clinical parameters assessment

Demographic and clinical parameters data of the Non-MetS and MetS groups, as well as the Non-IR and IR groups, are shown as mean ± SEM, including age, BW, BMI, WC, HC, WHR, %fat, fat mass, SBP, DBP, and MAP (Table 1). BW, %fat, and fat mass were assessed using the TANITA^®^ bioelectrical impedance analysis scale. WC was measured at the umbilicus during silent respiration in a standing position^81^, and HC was measured at the maximum circumference of the buttock^82^. SBP and DBP were measured in the supine position using a sphygmomanometer after a 30-minute bed rest.

### HRV measurement

The HRV measurement protocols were performed in a silent room, with temperature maintained at 23–25 °C and humidity at 50–60%, after a 12-hour overnight fast, according to our previous publication^83^. In the morning, at approximately 9.00 a.m., participants rested in bed for 15 min before undergoing a 15-minute electrocardiogram (ECG) recording in the supine position using the Finometer^®^ Pro device equipped with an ECG module (lead II). The ECG data were recorded at a 200 Hz sampling rate and analyzed using the BeatScope^®^ Easy program (Finapres Medical System BV, Amsterdam, The Netherlands). A 5-minute segment of stable ECG data, free of ectopic beats (normal-to-normal (NN) intervals), was selected for time- and frequency-domain HRV parameters using LabChart^®^8Pro software (ADInstruments, Castle Hill, Australia).

### Biochemical data analysis

Fasting blood samples were collected from the participants to evaluate the glucose metabolic factors (glucose and insulin), lipid profiles (plasma cholesterol, plasma TG, HDL-C, and LDL-C), and kidney functions tests (plasma BUN and Cr.). The blood samples were analyzed by the central laboratory of the Department of Clinical Pathology, Faculty of Medicine Siriraj Hospital, Mahidol University, Thailand, using enzymatic techniques for glucose, cholesterol, LDL-C, HDL-C, TG, BUN and Cr., and a sandwich immunoassay with electrochemiluminescence detection for insulin, following standardized protocols. Additionally, fasting glucose and insulin levels were used to further determine HOMA-IR, calculated as: (fasting glucose (mg/dL) x fasting insulin (µU/mL))/405^84^, and QUICKI calculated as: 1/((log (fasting insulin µU/mL) + log (fasting glucose mg/dL))^85^, to assess insulin resistance and insulin sensitivity, respectively. Cr. Cl was calculated using the Cockcroft-Gault equation as: Cr. Cl = ((140 - age) x BW)/(serum Cr. x 72) ​ for males or ((140 - age) x BW)/(serum Cr. x 72 × 0.85) ​ for females^86^. eGFR was calculated using the CKD-EPI equation as: eGFR = 141 x min (serum Cr. x 0.0113/κ,1)^α^ x max (serum Cr. x 0.0113/κ,1)^−1.209^ × 0.993^age^ x 1.018 [if female] x 1.159 [if black], where κ is 0.7 for females and 0.9 for males, α is −0.329 for females and − 0.411 for males, min indicates the minimum of serum Cr./κ or 1, and max indicates the maximum of serum Cr./κ or 1^87^.

### Hormonal assay

Serum leptin and adiponectin levels were determined using commercial enzyme-linked immunosorbent assay (ELISA) kits, while kisspeptin was analyzed with a commercial enzyme immunoassay kit (Phoenix Pharmaceuticals, CA, USA), following the manufacturer’s guidelines. The detection ranges were 0.313–20.313 ng/mL for leptin, 0–100 ng/mL for kisspeptin, and 0.15–10.15 ng/mL for adiponectin, with minimum detectable levels of 0.313 ng/mL, 0.05 ng/mL, and 0.15 ng/mL, respectively. Serum samples were diluted with assay buffer at ratios of 1:5 for leptin, 1:2 for kisspeptin, and 1:5,000 for adiponectin. The intra-assay and inter-assay variability were 6.66% and 6.74% for leptin, 6.38% and 7.85% for kisspeptin, and 2.33% and 3.55% for adiponectin. Optical density was measured at 450 nm using the Synergy HT Multi-Detection Microplate Reader (BioTek Instruments, Inc., Winooski, VT, USA).

### Statistical analysis

Statistical analysis was performed using the Statistics Package for the Social Sciences (SPSS) software version 29 (IBM Corporation, New York, USA). The Kolmogorov-Smirnov test was used to assess normality. Comparisons between the Non-MetS and MetS groups, as well as the Non-IR and IR groups, were performed using the independent-samples t-test for normally distributed data or a nonparametric test for non-normally distributed data. Effect sizes (Cohen’s *d*) were calculated to quantify the magnitude of group differences, and post hoc power analyses were conducted using the observed means and standard deviations to assess the strength of group comparisons. Correlations between two factors were determined using Pearson’s correlation for normally distributed data or Spearman’s correlation for non-normally distributed data. Since the correlations of SBP and DBP with glucose metabolic factors, lipid profiles, and hormonal factors were reported in our previous study^83^, we analyzed their correlations only with the number of MetS criteria and kidney function. Multiple linear regression analysis was used to determine which factors mainly contributed to the number of MetS criteria, serum leptin and adiponectin. All standard regression assumptions (normality, linearity, homoscedasticity, and collinearity) were tested and confirmed, with normality evaluated by histograms and P-P plots. A *p-*value of *< 0.05* was used to define statistical significance.

## Data Availability

The data that support the findings of this study are available from the corresponding author upon reasonable request.
